# Falling of older adults with cognitive impairment: a new home environment evaluation chart and a preliminary examination in the Wakuya Project

**DOI:** 10.1590/1980-5764-DN-2022-0009

**Published:** 2023-05-29

**Authors:** Tomohiro Sugawara, Keiichi Kumai, Miwako Shoji, Kenichi Meguro

**Affiliations:** 1 Tohoku University, New Industry Creation Hatchery Center, Geriatric Behavioral Neurology Project, Sendai, Miyagi, Japan.; 2 Tohoku University, Cyclotron Radioisotope Center, Sendai, Miyagi, Japan.; 3 Tohoku University Graduate School of Medicine, Sendai, Miyagi, Japan.

**Keywords:** Dementia, Cognitive Dysfunction, Home Environment, Demência, Disfunção Cognitiva, Ambiente Domiciliar

## Abstract

It is important to evaluate the home environment because most fractures in older adults are caused by falls indoors. However, previous studies have not separated individual abilities from environments. Also, the interrelationship between falls, cognitive function, and home environments has not been clarified.

**Objectives:**

The purpose of this study was to develop an evaluation method specific to the home environment and examine the effects of environment and cognitive impairment on falls.

**Methods:**

This was a case-control study analyzing 95 older adults living in the community. A Visiting Checklist for the Home Environment (VICHe) was developed and examined for reliability and validity. Inter-rater reliability (IRR) was examined by determining Cohen's kappa and the intra-class correlation coefficient. Guttman's split-half method was used for internal consistency, and Cronbach's alpha coefficient was obtained. Criterion-related validity was confirmed by Spearman's rank correlation coefficient with the Fall Risk Index's (FRI) total score of the environmental factor items. As a preliminary study, trends in the number of falls by cognitive function and home environment were examined.

**Results:**

The VICHe obtained validity, but the IRR was inadequate. In contrast, the version that focused on the on-floor environment (VICHe-OFI) showed IRR for all items and validity through correlations with the FRI. The number of fallers increased in the cognitive impairment group when the home environment was bad.

**Conclusions:**

Reliability and validity of the VICHe-OFI were obtained. Preliminary examination using this scale indicates that falls in the home of the elderly may be more affected by the home environment as cognitive function declines.

## RESUMO.

A avaliação do ambiente doméstico é importante pois a maioria das fraturas em idosos é causada por quedas dentro de casa. No entanto, estudos anteriores não separaram as habilidades individuais dos ambientes. Além disso, a inter-relação entre quedas, função cognitiva e ambientes domésticos não foi esclarecida.

**Objetivo:**

O objetivo foi desenvolver um método de avaliação específico para o ambiente doméstico e examinar os efeitos do ambiente e do comprometimento cognitivo nas quedas.

**Métodos:**

Este estudo foi um estudo de caso-controle analisando 95 idosos residentes na comunidade. Uma Lista de Verificação de Visitas para o Ambiente Doméstico *(Visiting Checklist for Home Environment* — VICHe) foi desenvolvida e examinada quanto à confiabilidade e validade. A confiabilidade entre avaliadores (CEA) foi examinada pela determinação do kappa de Cohen e do coeficiente de correlação intraclasse. O método *split-half* de Guttman foi usado para consistência interna e foi obtido o coeficiente alfa de Cronbach. A validade de critério foi confirmada pelo coeficiente de correlação de Spearman com a pontuação total da escala de risco de queda (ERQ) dos itens do fator ambiental. Como um estudo preliminar, foram examinadas as tendências no número de quedas por função cognitiva e ambiente doméstico.

**Resultados:**

O VICHe obteve validade, mas a CEA foi inadequada. Por outro lado, a versão que enfocou o ambiente “no chão” (VICHe-OFI) apresentou CEA para todos os itens e validade por meio de correlações com a ERQ. O número de quedas aumentou no grupo com comprometimento cognitivo quando o ambiente doméstico era ruim.

**Conclusões:**

Obteve-se confiabilidade e validade do VICHe-OFI. O exame preliminar com essa escala indica que as quedas do idoso no domicílio podem ser mais afetadas pelo ambiente doméstico à medida que a função cognitiva diminui.

## INTRODUCTION

Fall is a serious event that can lead to fractures, resulting in a decline in activities of daily living (ADL) and the need for nursing care. Tinetti identified sensory and motor disorders, dementia, previous falls, gait impairment, and depression as risk factors for falls^1,2^. However, there is still insufficient evidence for improving the home environment. Currently, physiotherapy and exercise are effective interventions for fall prevention, but there is no description of the evaluation of the home environment.

Toba et al. developed the Fall Risk Index (FRI)^3,4^ to identify significant risk factors for fall. The FRI is unique in assessing environmental factors; however, this assessment may still not be able to show an association between pure home environment factors and fall.

Cognitive impairment is a strong risk factor for fall^5,6^, but relationship with the home environment is not in agreement and there is no standardization for assessment. A systematic review identified five assessments that focus only on physical home environment hazards^7^. In eight previous studies^8–15^ using these five assessments, only two studies^8,14^ examined the relationship between home environment and fall but did not agree with the relationship.

Assessments for the home environment in Japan include the House Environment Checklist^16^, Housing Checklist for Fall Prevention^17^, WeHSA-J^18^, and the FRI and MDS-HC 2.0^19^, which partially include environmental assessment. Among these, only the FRI has shown evidence for falls, but it has limitations as a self-administered questionnaire and does not assess the individual's ability and environment separately.

Our research team has performed community-based studies using the Clinical Dementia Rating (CDR)^20,21^. Furthermore, in the Wakuya Project, while only a small number of healthy participants fell^22^, the number of multiple falls increased in dementia people and was associated with results on the Timed Up and Go Test (TUG), a motor function test, and the Trail Making Test part A (TMT-A) and Digit Symbol (DS) tests, which evaluate executive function.^23^

Previous literature does not provide an environmental evaluation scale that is independent of the environment and the person's ability to evaluate the environment with established evidence of a relationship to falls, and no studies have examined the interaction between the environment and cognitive function about falls.

The aims of this study were:

- To develop a home environment evaluation method independent of the individual's ability, and
- As a preliminary examination, to examine the effects of the environment and cognitive decline on falls.

## METHODS

### Participants and the Wakuya Project

The Wakuya Project is a contracted research study started in 2014 in Wakuya, Miyagi Prefecture, Japan, by the Tohoku University. The Wakuya Project targeted 2,112 residents of the East and West Model Districts (1,010 in the East District and 1,102 in the West District) out of a total of 2,839 older adults aged 75 years or older living in Wakuya Town. Of the residents in the model districts, 201 residents participated in the study, of whom 180 participated in the Dementia Risk Survey (DRS) and 21 in the Home Visit Survey (HVS).

In Japan, there is an official health checkup called a resident health checkup, which is always conducted by local governments based on the law, but this does not include screening for dementia. The participants of the DRS were recruited from the residents in the model districts by publicity, information sessions, and mailings, with an equal number of participants from the districts with low and high health checkup rates, respectively, based on the health checkup rates in each district. The residents were informed about the survey at a community center, and their consent was obtained. The 2018 and 2019 “Follow-up Visit” surveyed consenting DRS participants (57 in 2018; 21 in 2019) in detail about falls and the home environment.

In 2019, HVS was conducted to make up for the missing number of healthy groups (CDR 0) in the analysis results, and the survey was conducted in detail on falls and home environment. Participants were recruited from care prevention classes in the target area and outpatients of the Wakuya National Health Insurance Hospital by the staff of the Community Comprehensive Support Center (CCSC) and the outpatients of the Wakuya National Health Insurance Hospital. The data acquisition methods used in the present analysis were the same for the “Follow-up Visit” and the HVS.

**Figure 1:**
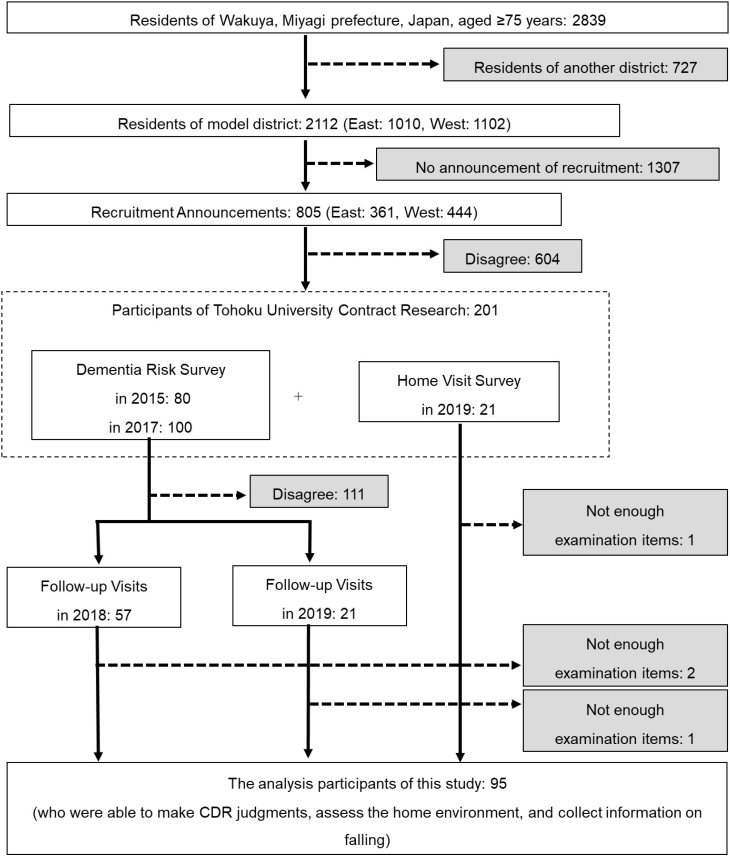
Selection of participants for the study. Of the 2,839 residents of Wakuya, Miyagi Prefecture, Japan, aged ≥75 years, 201 people (14.1%) participated in contract research performed by Tohoku University. Follow-up visits and home visit surveys to assess clinical dementia rating (CDR) and home environment were possible in 95 residents (3.3%), and these people were selected as participants in the current study.

Finally, of the participants who were able to complete the Follow-up Visit and HVS, analyses were conducted on 95 participants for whom CDR judgment, home environment assessment, and fall information were obtained (Figure 1). The data acquisition methods used in the present analysis were the same for the “Follow-up Visit” and the HVS.

### Assessment

Demographics and CDR results for cognitive and motor function tests associated with fall^1,2,5,23^ were extracted from the database of the Wakuya Project. Fall and the home environment were examined using the Visiting Checklist for the Home Environment (VICHe) developed in this study.

#### Visiting checklist for the home environment

The items on the VICHe were developed partially from the literature^17,24,25^ and based on the author's clinical experience as a physical therapist and care manager, with an original list of physical items that may be relevant to falls in the home. The key spaces are:

- Entrance approach,
- Entrance,
- Hallway,
- Toilet,
- Bedroom,
- Living room,
- Stairs,
- Bathroom,
- Dressing room,
- Washroom,
- Kitchen,
- Back door, and
- Balcony.

All 13 places include items common to all rooms and environmental items for each room, with a total of 98 items. For each item, 1 point is given if it is considered to be a risk for a fall. Information was collected by visiting the locations of falls in the past year and the rooms used or passed through from the time they woke up in the morning until they went to bed at night (Supplementary Material).

The VICHe was completed at 2018 follow-up visits or in the 2019 HVS by a physical therapist or nurse who accompanied a CCSC staff member to the participant's home to assess the home environment. To examine the reliability of the VICHe, 11 participants who gave consent were visited again by two investigators (two out of a physical therapist, nurse, and social worker) to check the home environment independently.

#### Clinical dementia rating

The CDR is an observational method used worldwide^20,26^. Information was collected by a public health nurse and a social worker, who were CCSC staff members who visited the participant's home and interviewed the person and their family on the participant's living conditions, based on a visitation questionnaire^27^. A decision on CDR was then made using the CDR worksheet at meetings attended by public health nurses and doctors.

#### History of fall

A 2-year history of fall at home was collected using a definition of fall in accordance with that in the FICSIT trial^28^ of an unintentional coming to rest on the ground, floor, or other lower level.

#### Motor function assessment, activities of daily living

Grip strength was measured with an M-type grip strength meter. The TUG was measured at a comfortable speed; a chair with a 40-cm seat was used. The 6-m walking speed was measured twice at a comfortable speed on a 1.5-m runway in front of and behind the 6-m walkway, and the mean was obtained. In the open-eyed one-leg standing test (OLS), the participant was asked to “stand with your hands on your hips and one leg about 5 cm above the floor for as long as possible.” The maximum time was set at 120 s. The Barthel index (BI) was used to assess the ADL. To examine the relationship between outdoor activities and fall at home, the level of physical activity (PA) was determined by exercise habits^29^. Data were obtained in a survey of participants or family members. The METs calculation was based on the Mets of PA Table^30,31^ and the estimated PA/day (METs) was calculated as (item METs × days × hours)/7.

#### Fall risk index

The FRI is a 21-item, self-administered questionnaire^3^ to assess the risk of fall. A score of ≤10 indicates a high risk of multiple falls and a decline in basic ADL^4^. In this study, the total score for environmental items on FRI was used to examine the validity of the VICHe. Participants with dementia were also expected to participate, and responses were obtained from family members.

### Analysis

#### Visiting checklist for the home environment

Two analyses were performed. First, all items were scored and examined for reliability and validity. Cohen's kappa coefficient was calculated based on the assessments of two examiners. Since the total score changes depending on the number of rooms used by the participants, the total score was divided by the number of rooms used to obtain the score per room, which was defined as the mean environmental score. The ICC for inter-observer reliability was calculated using these scores. For consistency, reliability coefficients were calculated using the odd-even method with Guttman's split-half method. A Cronbach's alpha coefficient was also calculated.

Regarding criterion-related validity, the FRI environmental items were used as the gold standard for environmental evaluation in this study, and the correlation between mean scores on the VICHe and scores on the FRI environmental items was confirmed using Spearman's rank correlation coefficient. This is because the FRI is the only scale for which evidence has been shown to be related to falls in the home. As the study included participants with cognitive impairments, we also correlated their scores on the family FRI environmental items with their mean VICHe environmental scores. The validity of the results was examined using a one-tailed test.

Second, a version of the VICHe focused on on-floor improvement (VICHe-OFI) was used. The VICHe-OFI was developed by extracting from the 98 items, only those items of “tripping ease” and “slipperiness” that can be solved by simple environmental improvements that do not involve construction work and those with a good Cohen's kappa coefficient. The reliability and validity of the VICHe-OFI were examined using the methods described above for the total VICHe.

#### Relationships among fall at home, cognitive impairment, and home environment

Participants with a history of fall in the past 2 years were classified as the fall group and those without this history were defined as the no fall group. Based on cognitive function, participants were also classified into two groups: a healthy group (CDR 0) and a group with cognitive impairment (CDR ≥0.5). The home environment was classified using the mean of the VICHe-OFI environmental scores.

Differences in the number of fallers between participants with good and bad home environments and between the healthy (CDR 0) and cognitive impairment group (CDR ≥0.5) were examined. In participants with cognitive impairment, the mean of each assessment item was compared for those with and without fall in the bad environment group. It was assumed that each item would decline in the fall group, and one-tailed tests were used for all comparisons, except for gender.

#### Statistical analysis

A Shapiro-Wilk test was used to confirm normality for all continuous variables before statistical analysis. For those with a normal distribution, a test for the mean (parametric test) was conducted, and for those without a normal distribution, a test for the difference in the distribution center (nonparametric test) was conducted. The significance level (risk rate) was set at p=0.05, and a Bonferroni correction was applied for multiple item tests. Cohen's kappa coefficient and the ICC were based on Landis and Koch^32^: 0.61–0.80 was judged to be substantial agreement, 0.81–1.00 as almost perfect or perfect agreement, and 0.61 as a cutoff value. Correlation coefficients were determined based on Guilford^33^, with r less than 0.020 being considered “slight almost negligible relationships”, 0.2–0.4 being “low correlation”, 0.4–0.7 being “moderate correlation”, and 0.7–0.8 being “very low correlation”; 0.7–0.9 is a high correlation (marked relationship) and 0.9 or more is a very high correlation (very dependable relationship).

### Ethics approval and consent to participate

This study was approved by the Ethics Committee of the Tohoku University Graduate School of Medicine (no. 2019-1-358). Consent was obtained on paper from all participants and their families.

## RESULTS

### Participant characteristics

Table 1 shows the demographics and motor functions of the participants. The number of rooms used by the 95 participants assessed by VICHe is as follows: 95 people used the approach, entrance, hallway, toilet, and living room; 94 used the bedroom; 42 used the stairs; 93 used the bathroom; 78 used the dressing room; 78 used the washroom; 86 used the kitchen; 43 used the back door; and 9 used the balcony.

**Table 1:** Demographics of the participants.

	n	Healthy group (CDR 0)	Cognitive impairment group (CDR ≥0.5)	Statistic, p-value
Number of participants (men/women)	95	23 (11/12)	72 (27/45)	χ^2^=0.774, p>0.05
Age, years	95	80.5 (3.8)	81.7 (4.2)	U=951.5, p>0.05
Years of education, years	93	11.7 (1.7)	10.4 (2.3)	U=524.0, p=0.008*
BI, scores	94	100 (0.0)	97.5 (6.7)	U=655.5, p=0.022*
MMSE, scores	94	25.4 (3.1)	22.5 (3.8)	U=452.0, p=0.001†
TMT-A, s	92	61.7 (19.4)	65.6 (28.9)	U=806.5, p>0.05
DS 90, correct answers	94	32.7 (9.3)	27.2 (10.2)	t=2.291, p=0.024*
DS 120, correct answers	94	43.2 (11.7)	36.8 (13.9)	t=2.004, p=0.048*
Grip strength, kg	93	24.0 (7.3)	21.0 (7.4)	t=1.777, p>0.05
Right OLS, s	93	27.1 (24.3)	12.9 (19.5)	U=376.0, p<0.001†
Left OLS, s	93	20.1 (21.5)	9.7 (14.9)	U=484.0, p=0.004†
6 mw, m/s	93	1.4 (0.2)	1.2 (0.3)	U=499.0, p=0.006*
TUG, s	93	8.7 (1.4)	11.3 (4.9)	U=1089.5, p=0.011*
FRI, scores	94	4.8 (3.0)	8.2 (3.8)	t=-3.975, p<0.001†
Estimated PA/day, METs	88	233.2 (243.8)	201.9 (256.8)	U=610.5, p>0.05
Daily activity at home, items	88	7.6 (3.1)	7.0 (2.8)	U=605.0, p>0.05
Number of rooms used, rooms	95	11.0 (0.8)	10.4 (1.3)	U=588.0, p=0.029*

Abbreviations: n: number of participants; CDR: clinical dementia rating; BI: Barthel index; MMSE: Mini-Mental State Examination; TMT-A: Trail Making Test A; DS 90: digit symbol 90 s; DS 120: digit symbol 120 s; OLS: open-eyed one-leg standing time; TUG: Timed Up and Go Test; FRI: Fall Risk Index; PA: physical activity.

* Notes: p<0.05;

† p<0.0036 (Bonferroni correction).

Data were compared between healthy participants (CDR 0) (n=23) and those with cognitive impairment (CDR ≥0.5) (n=72): gender by χ² test (not significant); age, years of education, MMSE, TMT-A, BI, 6-m walking speed, TUG, right OLS, left OLS, PA/day, daily activities at home, and number of rooms used by Mann-Whitney U test; and DS, grip strength, and FRI by two-sample t-test.

Years of education, MMSE, DS 90, DS 120, BI, right OLS, left OLS, 6-m walking speed, TUG, FRI, and number of rooms used were significantly lower in participants with cognitive impairment. A Bonferroni correction showed more significant decreases in MMSE, right OLS, left OLS, and FRI.

### Visiting checklist for the home environment

Cohen's kappa coefficient could be calculated for 51 of the 98 items on the VICHE and was ≥0.61 for 43 items (substantial agreement) (Supplementary Material). Table 2 shows the reliability and validity of the VICHe.

**Table 2:** Reliability and validity of the VICHe.

Visiting Checklist on Home Environment (VICHe)		All items (98 items)	On-floor improvement (OFI) (7 items)
Reliability	Cohen's kappa	κ>0.61: 43 items	κ=0.62–1.00
	ICC (2, 2)	p=0.97 (95%CI 0.88–0.99)	p=0.87 (95%CI 0.54–0.99)
	Guttman's split-half method	G=0.98	G=0.34
	Cronbach's α	α=0.62	α=0.45
Validity	Correlation with FRI environmental scores	r_s_=0.21*	r_s_=0.17
	Correlation with FRI family environmental scores	r_s_=0.18	r_s_=0.20*

Abbreviations: ICC: in-class correlation coefficient; CI: confidence interval; FRI: Fall Risk Index.

* Note: p<0.05.

### Relationships among fall at home, cognitive impairment, and home environment

#### Category of home environment

The median environmental score of 0.14 for all participants was used as a criterion for operative classification into two groups with a good environment (n=21, score <0.14) and a bad environment (n=74, score ≥0.14).

#### Fall at home based on cognitive impairment and home environment

Table 3 shows the comparison of each test result based on fall history. The fall group showed more significant decreases in age, BI, OLS, 6-m walking speed, TUG, and FRI (p<0.0036).

**Table 3:** Comparison of each test result based on fall history.

	n	No fall	Fall	Statistic	p-value
Number of participants (men/women)	95	67 (29/38)	28 (9/19)	χ^2^=1.021	p=0.312
Age, years	95	80.6 (3.8)	83.5 (4.0)	U=1319.0	p=0.002*
Years of education, years	93	10.7 (2.2)	10.7 (2.2)	U=833.0	p=0.729
BI, scores	94	99.0 (4.8)	96.1 (7.6)	U=653.0	p=0.000*
MMSE, scores	94	23.6 (3.8)	22.5 (3.9)	U=770.5	p=0.203
TMT-A, s	92	61.5 (22.8)	72.2 (33.8)	U=1017.0	p=0.232
DS 90, correct answers	94	30.3 (10.1)	24.5 (9.1)	t=2.573	p=0.012†
DS 120, correct answers	94	40.5 (13.5)	33.4 (12.8)	t=2.362	p=0.020†
Grip strength, kg	93	22.9 (6.8)	18.9 (7.7)	t=2.541	p=0.013†
Right OLS, s	93	20.7 (24.0)	6.6 (8.5)	U=454.0	p=0.000*
Left OLS, s	93	15.5 (19.6)	4.8 (5.1)	U=557.0	p=0.003*
6 mw, m/s	93	1.3 (0.3)	1.0 (0.4)	U=431.0	p=0.000*
TUG, s	93	9.5 (2.7)	13.4 (6.3)	U=1316.5	p=0.001*
FRI, scores	94	6.5 (3.5)	9.5 (4.0)	t=-3.624	p=0.000*
Estimated PA/day, METs	88	240.8 (262.9)	126.7 (205.2)	U=516.0	p=0.018†
Daily activity at home, items	88	7.3 (2.8)	6.8 (3.1)	U=672.5	p=0.368
Number of rooms used, rooms	95	10.7 (1.2)	10.1(1.3)	U=690.0	p=0.034†

Abbreviations: n: number of participants; BI: Barthel index; MMSE: Mini-Mental State Examination; TMT-A: Trail Making Test A; DS 90: Digit Symbol 90 s; DS 120: digit symbol 120 s; OLS: open-eyed one-leg standing time; TUG: Timed Up and Go Test; FRI: Fall Risk Index; PA: physical activity.

* Notes: p<0.0036 (Bonferroni correction);

† p<0.05.

Data are shown as mean (SD) values. There was no significant difference in gender between the no fall (n=67) and fall (n=28) groups, but the fall group had significantly lower age, DS 90, DS 120, BI, right OLS, left OLS, 6-m walking speed, TUG, FRI, estimated PA/day, and number of rooms used. A Bonferroni correction showed more significant decreases in age, BI, right OLS, left OLS, 6-m walking speed, TUG, and FRI.

Table 4 shows a comparison of test results for participants with cognitive impairment and bad home environment.

**Table 4:** Comparison of each test result for participants with cognitive impairment and bad home environment.

	n	No fall	Fall	Statistic	p-value
Number of participants (men/women)	53	30 (12/18)	23 (7/16)	χ^2^=0.518	p>0.05
Age, years	53	81.3 (3.7)	83.4 (4.3)	U=443.5	p=0.038
Years of education, years	51	9.9 (2.4)	10.7 (2.4)	U=361.5	p>0.05
BI, scores	52	99.0 (4.7)	95.4 (8.2)	U=227.0	p=0.0030*
MMSE, scores	53	22.4 (4.2)	22.6 (3.8)	U=356.0	p>0.05
TMT-A, s	51	61.7 (17.6)	71.0 (27.7)	U=377.0	p>0.05
DS 90, correct answers	53	27.6 (11.1)	23.8 (8.9)	t=-1.333	p>0.05
DS 120, correct answers	53	37.0 (14.8)	32.9 (13.0)	t=-1.051	p>0.05
Grip strength, kg	52	21.6 (6.0)	18.3 (7.9)	t=-1.745	p=0.043
Right OLS, s	52	12.2 (17.7)	6.8 (9.1)	U=259.0	p>0.05
Left OLS, s	52	7.7 (10.4)	5.1 (5.5)	U=284.0	p>0.05
6 mw, m/s	52	1.2 (0.3)	1.0 (0.4)	U=186.5	p=0.0035*
TUG, s	52	10.7 (3.3)	13.8 (6.3)	U=440.0	p=0.025
FRI, scores	53	7.5 (3.7)	9.8 (4.1)	t=2.159	p=0.018
Estimated PA/day, METs	47	256.4 (296.9)	122.6 (215.8)	U=162.5	p=0.012
Daily activity at home, items	47	7.1 (2.6)	6.7 (3.0)	U=221.5	p>0.05
Number of rooms used, rooms	53	10.6 (1.4)	10.0 (1.3)	U=264.0	p=0.018

Abbreviations: n: number of participants; BI: Barthel index; MMSE: Mini-Mental State Examination; TMT-A: Trail Making Test A; DS 90: Digit Symbol 90 s; DS 120: digit symbol 120 s; OLS: open-eyed one-leg standing time; TUG: Timed Up and Go Test; FRI: Fall Risk Index; PA: physical activity.

* Note: p<0.0036 (Bonferroni correction).

Data are shown as mean (SD) values. One-tailed test was used for all items except gender.

A comparison is shown for participants with cognitive impairment (CDR ≥0.5) and a bad home environment who did not fall (n=30) and did fall (n=23). There was no significant difference in gender between the groups. The fall group had a significantly lower BI and 6-m walking speed.

**Figure 2:**
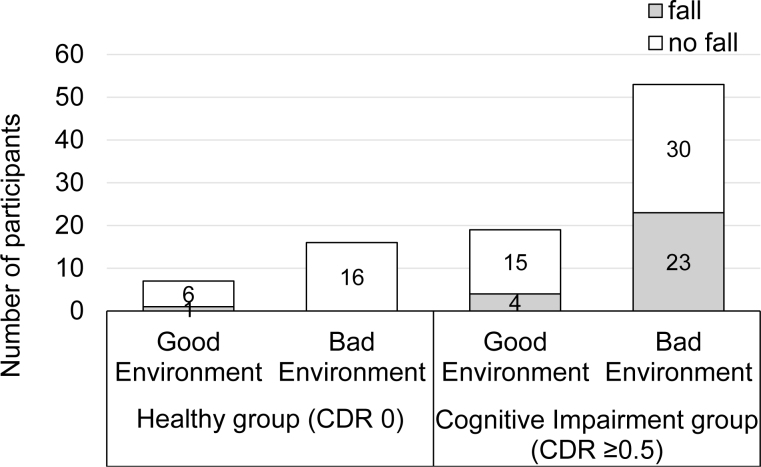
The number of fallers by cognitive impairment and home environment. Only one healthy participant (clinical dementia rating [CDR] 0) had a fall at home in a good environment. Participants with cognitive impairment (CDR ≥0.5) had 4 falls in a good environment and 23 falls in a bad environment.

Figure 2 illustrates the number of fallers by cognitive impairment and home environment.

## DISCUSSION

### Reliability and validity of the VICHe

Reliability of VICHe (all items) was shown by Cohen's kappa coefficient for only 43 of the 98 items. This may be because only 11 participants had revisits for environmental assessment to examine the reliability, in contrast to the large number of items to be checked. In contrast, reliability based on Cohen's kappa coefficient was obtained for all items on the VICHe-OFI. The ICC based on the mean environmental scores among examiners was also good. Therefore, we consider that the reliability of the assessment was shown.

The validity of the VICHe-OFI could be considered valid because it correlated with the scores of the FRI family environment items. This suggests that older adults may be less aware of the danger of the on-floor environment than family members. Therefore, it may be necessary for family members who live with an elderly person to pay attention to the on-floor environment and to clean up the floor.

### Relationships among fall at home, cognitive impairment, and home environment

People with cognitive impairment and a bad on-floor environment tended more likely to fall. Even after controlling for cognitive function and home environment, decreases in BI and 6-m walking speed were significant risk factors for fall. This result is consistent with previous studies^1,2,23,29^.

Participants with fall, cognitive impairment, and a bad home environment showed a significant decrease in BI and 6-m walking speed compared to those without fall in the same groups. Interestingly, there was no difference in executive function, as indicated by the TMT-A and DS, and only a decline in ADL and gait function significantly affected fall. For a participant with cognitive impairment and executive functions in decline, if there is an additional decline in gait and movement ability, executive functions may be overloaded by the need to pay more attention to avoidance of fall when moving around the home and the need to distribute attention. This may result in inadequate attention to hazardous environments, which may increase the risk of fall.

### Application to medical and nursing care

The VICHe-OFI, which has been shown to be reliable and valid, may be useful in assessing the risk of falls in older adults due to their home environment. In addition, because the VICHe-OFI is an evaluation of the environment independent of the individual's ability and can be scored, it is well suited to examining the interrelationships between factors such as motor and cognitive function and the environment. The VICHe (all items) takes about 15–20 min, which may be too long for a physician to perform. In addition, since the items are designed for Japanese houses, it may be necessary to reconsider the items to be used in other countries with different housing cultures. However, since the VICHe-OFI is a scale specific to the on-floor environment, the time required is greatly reduced to about 5 min, and the content is the presence or absence of cords, rug snagging, etc., it may be allowed to be generic in homes in other cultures.

In the application of the results of this study to the prevention of fall in older adults living in the community, two points are important in reducing the risk of fall:

- Early detection of cognitive impairment and early intervention and
- Improvement of the floor environment through tidying.

### Study limitations

One of the limitations of this study is that all houses in Wakuya are owner-occupied, and most are old rural Japanese houses. In addition, because few participants were certified as needing nursing care, few were barrier-free. This suggests a bias in the target population. To examine the effects of steps and handrails, it will be necessary to examine other areas, including urban areas and condominium households. The VICHe items are listed by the author originally based on references, so there may be room for further opinions to be reflected in the items.

As is clear from the previous study, only a small number of the healthy group fell, and the preliminary examination, a comparison of the number of fallers, made it difficult to obtain sufficient statistical evidence due to the insufficient sample size of the healthy group.

We developed an original VICHe. The reliability and validity of the VICHe were shown using a version focused on on-floor improvement. Preliminary examination using this scale indicates that falls in the home of the elderly may be more affected by the home environment as cognitive function declines. Improvement of home environments may be necessary to prevent falls.
